# A Prognostic Gene Expression Profile That Predicts Circulating Tumor Cell Presence in Breast Cancer Patients

**DOI:** 10.1371/journal.pone.0032426

**Published:** 2012-02-23

**Authors:** Timothy J. Molloy, Paul Roepman, Bjørn Naume, Laura J. van't Veer

**Affiliations:** 1 Division of Experimental Therapy, The Netherlands Cancer Institute, Amsterdam, The Netherlands; 2 Agendia BV, Amsterdam, The Netherlands; 3 Department of Oncology, Oslo University Hospital, The Norwegian Radium Hospital, Oslo, Norway; Baylor College of Medicine, United States of America

## Abstract

The detection of circulating tumor cells (CTCs) in the peripheral blood and microarray gene expression profiling of the primary tumor are two promising new technologies able to provide valuable prognostic data for patients with breast cancer. Meta-analyses of several established prognostic breast cancer gene expression profiles in large patient cohorts have demonstrated that despite sharing few genes, their delineation of patients into “good prognosis” or “poor prognosis” are frequently very highly correlated, and combining prognostic profiles does not increase prognostic power. In the current study, we aimed to develop a novel profile which provided independent prognostic data by building a signature predictive of CTC status rather than outcome. Microarray gene expression data from an initial training cohort of 72 breast cancer patients for which CTC status had been determined in a previous study using a multimarker QPCR-based assay was used to develop a CTC-predictive profile. The generated profile was validated in two independent datasets of 49 and 123 patients and confirmed to be both predictive of CTC status, and independently prognostic. Importantly, the “CTC profile” also provided prognostic information independent of the well-established and powerful ‘70-gene’ prognostic breast cancer signature. This profile therefore has the potential to not only add prognostic information to currently-available microarray tests but in some circumstances even replace blood-based prognostic CTC tests at time of diagnosis for those patients already undergoing testing by multigene assays.

## Introduction

Approximately one third of all women with primary breast cancer will eventually develop metastatic disease, which represents the final step in the progression of malignancy and is the primary cause of mortality among cancer patients. Metastasis occurs as a result of the movement of a single, clinically occult micrometastatic cell from the primary tumor through the blood or lymphatic system to a remote site, where it lodges and begins to proliferate. The detection of these circulating tumor cells (CTCs) during their migration is of prognostic significance, with the presence of CTCs in the blood correlated with significantly poorer survival in both early-stage and metastatic breast cancer patients [1], [2], [3]. In addition to the prognostic value of CTC detection, it can also be used as a tool to measure systemic treatment response – Xenidis, *et al.* demonstrated that cytokeratin-19 (CK-19) mRNA-positive circulating tumor cells (CTCs) detection in the peripheral blood of women with early-stage breast cancer following chemotherapy was a significant predictor of treatment success, with patients in which CTCs remained after chemotherapy having more frequent clinical relapses and a poorer overall survival [4].

The use of microarray technology to profile gene expression in primary breast tumors has also resulted in the development of several powerful tests capable of predicting outcome in cancer [5], [6], [7], [8], [9], [10]. The vast majority of these have used supervised classification systems in which gene expression data paired with survival information are used as inputs to build signatures that are used to predict patient outcome. Interestingly, while these numerous prognostic profiles only rarely have genes in common, their classification of patients into risk categories in fact tend to be very highly correlated, suggesting that although different signatures may not include many of the same genes, they are almost certainly looking at the same limited number of biological pathways and processes [11], [12]. We have observed that CTC status and the ‘70-gene’ prognostic profile (currently commercially available as the MammaPrint test) provide independent prognostic information. In the current study, we therefore aimed to improve upon the prognostic power of existing gene expression profiles for breast cancer by building a gene signature predictive of CTC status. We hypothesized that by developing a profile that specifically predicts a tumors propensity to disseminate cells rather than patient outcome in general, we could to identify important gene networks that might otherwise not be found in current profiles but were nonetheless prognostic. Such an approach may increase the power of currently-available gene signatures for breast cancer.

## Materials and Methods

The methods and data described herein adhere to the REMARK criteria for the reporting of tumor marker prognostic studies [13], and the MIAME criteria for the reporting of microarray studies [14].

### Ethics statement

Written informed consent was obtained from all participants and the studies were approved by the Medical Ethical Committees of the Netherlands Cancer Institute and/or The Norwegian Radium Hospital.

### Patients groups

#### Training cohort

In two previous studies [2], [3], CTC status was determined prior to therapy (median 14 days after diagnosis) using multi-marker QPCR-based CTC assays (using tumour markers TFF1, TFF3, KRT19 and EPCAM in [2] and TFF3, SCGB2A2, KRT19 and EPCAM in [3]) at time of diagnosis for 192 women with stage I–IV breast cancer. These patients were recruited from the outpatient clinic of The Netherlands Cancer Institute/Antoni van Leeuwenhoek Hospital between May 2005 and May 2006. Fresh frozen primary tumor material from 72 of these patients (29 CTC-positive and 43 CTC-negative) was collected for micorarray analysis. All tumor specimens used were confirmed to contain at least 50% tumour cells. The median follow-up time for these patients was 37.4 months (Table 1). This microarray data is publically available in NCBI's GEO database under accession number GSE31364 and the corresponding clinical data is available in Table S3.

**Table 1:** Patient characteristics of the three cohorts used in CTC profile discovery and validation.

Characteristic	Group	Training Cohort	Validation Cohort 1	Validation Cohort 2
	Total number	72	49	123
Age	<40	5 (6.9%)	6 (12.2%)	32 (26.0%)
	40–50	22 (30.6%)	5 (10.2%)	71 (57.7%)
	50–60	28 (38.9%)	11 (22.4%)	20 (16.3%)
	60+	17 (23.6%)	27 (55.1%)	0 (0%)
Tumor Size	pT1 (≤20 mm)	47 (65.3%)	25 (51%)	48 (39.0%)
	pT2 (20–50 mm)	17 (23.1%)	22 (44.9%)	73 (59.4%)
	pT3 (>50 mm)	8 (11.1%)	0 (0%)	2 (1.6%)
	Unknown	0 (0%)	2 (4.1%)	0 (0%)
Histological grade	I	15 (20.8%)	8 (16.3%)	30 (24.4%)
	II	37 (51.4%)	28 (57.1%)	37 (30.1%)
	III	18 (25.0%)	13 (26.5%)	56 (45.5%)
	Unknown	2 (2.8%)	0 (0%)	0 (0%)
HR status	Negative	12 (16.7%)	17 (34.7%)	40 (32.5%)
	Positive	58 (80.6%)	31 (63.3%)	83 (67.5%)
	Unknown	2 (2.8%)	1 (2.0%)	0 (0%)
HER2/NEU status	Negative	54 (75%)	38 (77.6%)	93(75.6%)
	Positive	16 (22.2%)	8 (16.3%)	22 (17.9%)
	Unknown	2 (2.8%)	3 (6.1%)	8 (6.5%)
CTC status	Positive	31 (43.1%)	8 (16.3%)	Not Determined
	Negative	41 (56.9%)	41 (83.7%)	
CTC profile	Positive	29 (40.3%)	18 (36.7%)	81 (65.9%)
	Negative	43 (59.7%)	31 (63.3%)	42 (34.1%)
MammaPrint	High-risk	Not Determined	Not Determined	62 (50.8%)
	Low-risk			60 (49.2%)

#### Validation cohort 1

Microarray gene expression data from an independent cohort of 49 early-stage lymph-node negative breast cancer patients generated in a previous study [15] was used to validate the prediction accuracy and prognostic power of the CTC-predictive profile. These patients also had their CTC status determined using the same assay as for the training cohort [16] (using tumour markers TFF3, SCGB2A2, KRT19 and EPCAM), and have been previously described [17]. 15 of these patients underwent systemic treatment, including 3 patients given adjuvant chemotherapy, 11 given hormonal therapy, and 1 patient given both chemotherapy and hormonal therapy. 18 patients were CTC-negative, and 31 were CTC-positive. The median follow-up time for these patients was 9.2 years (Table 1). This microarray data is publically available in NCBI's GEO database under accession number GSE3985 and the corresponding clinical data (including predicted CTC status) is available in Table S2.

#### Validation cohort 2

mRNA from primary breast tumor material previously used to generate a publicly-available microarray expression dataset [8] was recently rehybridized onto newer, higher density whole-genome microarrays (as per [18], which describes the rehybridization of this material onto mini-arrays, which was performed at the same time). Microarray data from 123 early-stage lymph-node negative patients from this dataset, representing the lymph-node negative patient samples that were available for rehybridization from the original 151-patient cohort, was used for a second independent validation of the prognostic power of CTC-predictive profile and for comparison to the MammaPrint 70-gene prognostic profile for breast cancer [8], [9] (Table 1). 10 of these patients underwent systemic adjuvant chemotherapy, and the median follow-up time for these patients was 9.8 years. This microarray data is publically available athttp://microarray-pubs.stanford.edu/wound_NKI/explore.html, and the samples from this dataset used are listed in Table S4.

### Microarray analysis of training cohort

Whole-genome gene expression analysis of 72 primary breast tumor samples from the training patient cohort was performed on Agilent 44 k whole-genome microarrays. RNA isolation, amplification, Cy-dye labelling and hybridization was performed as described previously [19]. Breast tumor samples were hybridized in a duplicate dye-swap manner against a breast cancer reference pool that had been used previously to identify a prognostic breast cancer signature [9]. Microarrays were scanned and analysed using the Agilent Feature Extraction software (Agilent Technologies Inc, Santa Clara, CA). Gene expression data was corrected for background intensities and lowess-normalized. Duplicate dye-swap hybridizations were combined and used for the identification of a CTC-predictive gene expression profile.

### Identification of a CTC-predictive gene profile

A supervised training procedure using the microarray expression data from the training cohort was used to identify a profile that corresponded with CTC status. A 4-fold cross validation (CV) procedure within a leave-one-out (LOO) cross validation loop was used to determine the optimal set of profile genes and the unbiased profile performance on the training cohort. Within this CV procedure, genes were scored for their association with the CTC status (Student's T-statistic). A set of 34 genes that were used in all CV iterations was used for designing a ‘nearest-mean’ CTC-profile in a similar fashion to that previously described [9], [20]. The classification threshold was chosen for high overall accuracy and optimal positive predictive value to accurately identify tumors derived from CTC-positive patients.

The CTC-profile was subsequently validated on an independent microarray gene expression dataset from 49 early-stage lymph-node negative breast cancer tumors. These had been previously assayed using a custom cDNA microarray platform (consisting of 42,000 features representing 24271 unique cluster Ids; UniGene BuildNumber 173) produced at the Stanford Functional Genomics Facility (http://www.microarray.org/sfgf/jsp/home.jsp), and hybridised at an independent laboratory (Radium Hospital, Oslo) [15]. This data is publically available in the NCBI's GEO database as GEO dataset GSE3985. Validation was performed using 22 of the 34 CTC-profile genes that could be mapped to the cDNA microarrays. The prognostic value of this approximated CTC-profile was also determined using this cohort.

A second independent validation of the prognostic value of the CTC-predictive array-based profile was also performed using micorarray data from 123 early-stage lymph-node negative breast cancer patients from a second publicly-available microarray dataset [8] [18].

Finally, the partial 22-gene CTC profile used in the first validation cohort was also tested for predictive and prognostic significance in the both training and second validation dataset.

### Functional annotation and network analysis of CTC-profile genes

Functional annotation of the CTC-profile genes was performed using Ingenuity Pathway Analysis (IPA) software (Ingenuity Systems Inc, Redwood City, CA). Statistical significance for enrichment of functional groups within the set of 34 CTC-genes was based on Fisher exact test and corrected for multiple testing. Network analysis was performed using IPA and included 32 of the 34 CTC-genes (two genes could not be mapped by the IPA software).

### Statistics

Gene expression data analysis and statistical analysis was performed in R software with additional Bioconductor packages (www.r-project.org and www.bioconductor.org). Survival analysis was based on Cox proportional hazard models and censored for events not related to breast cancer progression. Classifier performance was determined by measuring the area under the Receiving Operating Characteristics (ROC) curve (AUC). The optimal classifier threshold was determined on the 72 training samples and subsequently applied to both independent validation cohorts. All measurements were associated with 95% confidence intervals (95% CI) and statistical tests were considered significant if *p*<0.05.

## Results

### CTC-predictive profile

**Figure 1:**
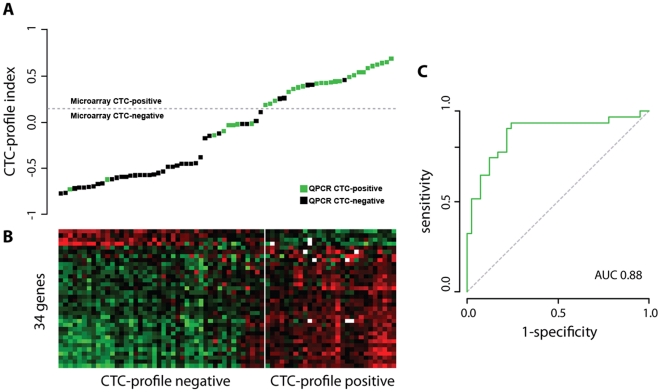
A gene expression profile derived from the primary tumor accurately predicts the presence of CTCs in the peripheral blood in breast cancer patients. (A) The CTC-profile indexes of 72 breast tumor samples are highly correlative with CTC status. Samples are ordered according to CTC-profile index and colored based on CTC-status. The dashed line indicates the classification threshold with optimal sensitivity and specificity. (B) A heatmap shows the level of expression of the 34 CTC profile genes for CTC-negative and CTC-positive patients. (C) The ROC curve of CTC-profile indexes compared to actual CTC status.

A multi-marker QPCR-based assay was previously used to determine circulating tumor cell (CTC) status from the peripheral blood samples of 192 breast cancer patients [2], [3]. To develop a gene expression profile that could predict CTC status, we analyzed the primary tumors of 72 of these patients using full-genome Agilent 44 K microarrays. A 4-fold cross validation (CV) procedure was used to determine the optimal set of genes expressed in the primary tumor that were associated with CTC status. We identified a set of 34 genes which formed the CTC-predictive profile (Table S1). The CTC-profile showed a significant leave-one-out CV performance of 82% for the prediction of CTC status (Figure 1) with an area under the Receiver Operating Characteristics (ROC) curve (AUC) of 0.88 and an optimal sensitivity of 74% (95%CI: 62%–82%) and specificity of 88% (95%CI: 79%–94%).

### Independent validation and prognostic significance of the CTC-predictive profile

**Figure 2:**
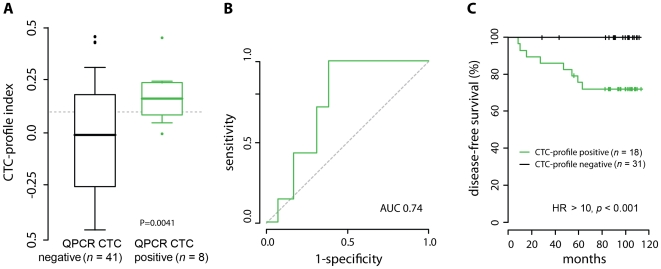
Independent validation of the CTC-predictive profile on 49 early-stage lymph-node negative breast cancer patients. (**A**)Distribution of CTC-profile indexes for CTC-positive and CTC-negative patients. The dashed line indicates the threshold as was determined in the training cohort. (**B**) Validation ROC curve showing classification performance of the CTC-profile. (**C**) Kaplan-Meier survival curves of relapse-free survival of patients classified as CTC-positive or CTC-negative using the CTC-profile.

Validation of the CTC-predictive profile was performed using micorarray gene expression data from an independent cohort of 49 lymph node-negative breast cancer patients for which CTC status had been previously determined using the same CTC detection assay as in the training cohort [16]. This independent validation dataset was generated using custom cDNA microarrays, on which 22 of the 34 CTC-profile genes were present. Despite one-third of the profile genes being missing on the platform, an approximated CTC-profile index was calculated for each sample (based on the threshold that was determined optimal on the training cohort). The missing genes resulted in the lower classification accuracy of 67.3%, with a sensitivity of 62.5% and a specificity of 68.3%. The distribution of profile index scores was significantly different for CTC-negative and CTC-positive patients (*p* = 0.004, Student's T-test), with a median index of −0.005 and 0.175, respectively (Figure 2A), and an AUC value of 0.74 (Figure 2B). For comparison, this partial 22-gene CTC profile was also used for classification in the training dataset. The correlation in classification between the full 34-gene and partial 22-gene CTC profile in the training dataset was very strong (r^2^ = 0.989), with an ROC AUC of 0.873 (versus 0.876 for the full profile).

Importantly, the classification of patients using the approximated CTC-profile was prognostic (hazard ratio (HR)>10 (*p*<0.001)), with the patients classified as CTC profile-negative having 100% relapse-free survival over the median 9.6-year follow-up period, versus 73% for CTC profile-positive classified patients (Figure 2C). Furthermore, multivariate analyses demonstrated that classification by the CTC profile was independent of other common clinical variables, such as tumor size, tumor grade, hormone receptor status, HER2/NEU status, and age (Table 2A) in this cohort.

**Table 2:** Multivariate Cox regression analysis including the CTC and MammaPrint profiles in addition to common clinical variables in the two validation cohorts of early-stage breast cancer patients.

	A: Validation dataset 1 (*n* = 49)		B: Validation dataset 2 (n = 123)	
Clinical Variable	HR (95% CI)	*p*-value	HR (95% CI)	*p*-value
CTC profile	>10	<0.001	2.8 (1.2–6.7)	0.022
MammaPrint	Not determined		5.4 (2.0–14.4)	0.001
Age (<50 years)	1.6 (0.3–9.2)	0.573	1.6 (0.5–5.0)	0.421
Tumor size (>2 cm)	2.4 (0.3–19.2)	0.397	1.3 (0.6–2.6)	0.513
Grade (>2)	2.4 (0.4–13.4)	0.320	1.5 (0.8–2.7)	0.159
ER status	4.7 (0.5–42.2)	0.168	0.6 (0.2–1.8)	0.365
PR status			0.7 (0.3–2.0)	0.533
HER2 status	0.4 (0.03–4.7)	0.434	1.0 (0.8–1.2)	0.893

Both profiles provide independent clinical information with respect to disease-free survival.

### Second independent validation and comparison to 70-gene profile

**Figure 3:**
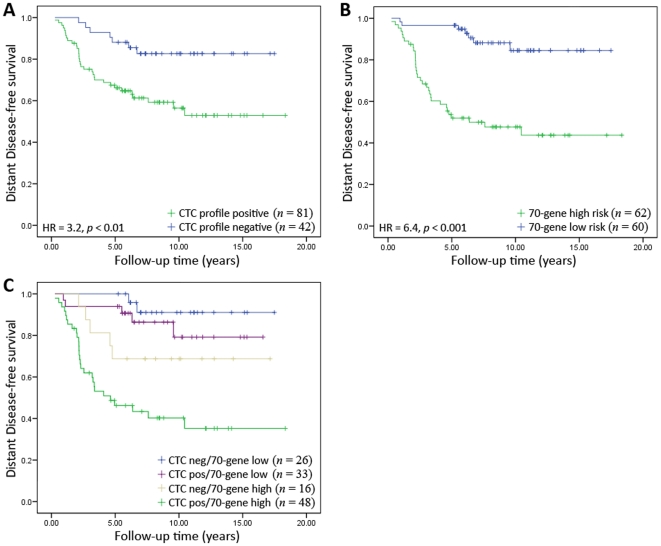
Kaplan-Meier survival analysis of a second independent validation patient cohort consisting of 123 early-stage breast cancer patients from the van de Vijver [8], [18] dataset classified with the CTC-profile (**A**), MammaPrint 70-gene profile (**B**), and both classifications combined (**C)**.

Further independent validation of the prognostic power of the CTC-profile was performed using publically-available microarray data from the primary tumors of 123 early-stage lymph-node negative breast cancer patients [8]. CTC-profile classification was determined using 33 of the 34 profile genes present on the array platform. Classification by the CTC-profile showed a HR of 3.16 (*p* = 0.006), confirming the prognostic value of the identified gene set. Patients with a CTC-negative profile had a 5-year disease-free survival (DFS) of 88% and a 10-year DFS of 83% versus 65% and 53%, respectively, for patients with a CTC-positive profile (Figure 3A). For comparison, the partial 22-gene CTC profile was also validated in this dataset. The correlation in classification to the 33-gene CTC profile was again high (r^2^ = 0.968), though it had somewhat lower prognostic value (HR = 2.56, *p* = 0.018).

We next investigated whether the CTC-profile added any additional independent prognostic information to the well-established, commercially available, 70-gene “MammaPrint” prognostic profile [9], [18]. Because more than half of the ‘70-gene’ classifier genes were missing on the arrays of the first independent validation dataset this was performed on the second validation dataset only. As previously reported [8], prognostic classification based on the 70-gene MammaPrint profile was highly significant in this patient group with a HR of 6.04 (*p*<0.001) and a 5-year and 10-year DFS of 95% and 84% for MammaPrint low-risk patients, versus 52% and 44% for MammaPrint high-risk patients (Figure 3B). When the classification results of both profiles were combined, risk classification was improved, with the patients classified as low-risk by both profiles having a 100% 5-year DFS and 91% 10-year DFS, and those patients classified as high-risk by both profiles having a 46% 5-year DFS and 35% 10-year DFS (Figure 3C). A multivariate analysis confirmed that despite the strong prognostic power of the MammaPrint profile, the CTC-profile added prognostic information with respect to disease-free survival, which was not only independent of the MammaPrint test, but also independent of tumor size, tumor grade, hormone receptor status, HER2/NEU status, and age (Table 2B).

### Functional annotation

**Figure 4:**
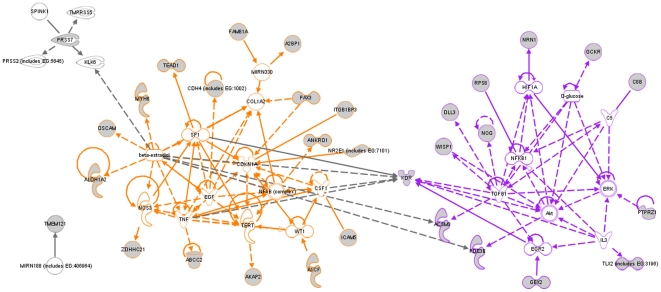
Network analysis of CTC-profile genes indicted two main functional networks: a cellular survival/proliferation associated network (colored orange) and a cellular migration/angiogenesis related network (colored purple). CTC-profile genes are indicated by grey symbols.

Functional annotation and gene network analysis demonstrated two main networks comprising 29 of the 34 CTC-profile genes (Figure 4). One network (16 genes) was enriched for genes associated with cellular survival and proliferation (*p* = 0.001), and included NOG, KDR, and ANKRD1. The second network (14 genes) included genes important for cellular migration (*p*<0.001) and angiogenesis (*p*<0.01). In addition, gene networks associated with cellular migration and adhesion made up a large proportion of the profile which was expected due to the specific aim of the study. These gene networks included MYH6, ICAM5, KDR, CDH4, and AKAP5, which are important for regulating cellular elongation and filopodia extension to enable cellular mobility, as well as NR2E1 whose gene product interacts with the fibronectin matrix during cellular migration, in addition to WISP1 and PAX3 which have been implicated in the epithelial-to-mesenchymal (EMT) transition important for the distant spread of micrometastatic tumor cells. Of the remaining four genes, two genes were outside these networks, and two genes were not annotated.

## Discussion

We and others have previously shown that the detection of CTCs in the peripheral blood of metastatic breast cancer patients is a significant predictor of poor overall survival [3]. Likewise, the detection of CTCs in the peripheral blood [21], or disseminated tumor cells (DTCs) in the bone marrow [22] or lymph nodes [23] in early-stage breast cancer patients is also predictive of poor outcome. Microarray studies have demonstrated that a tumors capacity to metastasize is apparent from very early on in its development [9], and this is consistent with the observation that CTCs can also be detected in breast cancer patients at the earliest stages of their disease [2]. We therefore hypothesized that microarray analyses of primary tumor material could be used to generate a profile specifically predictive of a tumors' propensity to disseminate cells. Such a profile could supplant the need for a separate prognostic CTC assay in certain circumstances, such as at the time of diagnosis for those patients already undergoing prognostic microarray testing. (CTC detection would remain useful in other roles however, such as monitoring adjuvant treatment response, or measuring changes in CTC levels over time.) We further hypothesized that such a prognostic gene expression signature may target pathways that may not otherwise be represented in current prognostic microarray profiles, and may therefore provide additional prognostic information independent of them. Finally, such a profile may prove to be a valuable source of CTC marker genes that could be useful in other studies.

We previously developed a highly sensitive and specific CTC detection platform combining a dual-antigen immunomagnetic tumor cell enrichment process with a multi-marker QPCR-based tumor cell detection assay [24], which was used to detect CTCs in two prospectively-collected breast cancer patient series [2], [3]. In the current study, we used whole-genome microarrays to quantify gene expression in the primary tumors of 72 of these patients, which led to the discovery of a 34-gene profile predictive of CTC status (Table S1). The 34-gene ‘CTC profile’ demonstrated a classification accuracy of 82% in this training cohort, and when approximated using only 22 profile genes in a second, independent microarray dataset [15] achieved a classification accuracy of 67%.

While high classification accuracy is desirable, ultimately the value of such a profile is determined by its prognostic power. We therefore next investigated whether the CTC-profile was prognostic in this and a third microarray dataset of 123 early-stage breast cancer patients [8]. It can be observed that more samples (66%) were predicted to be from CTC-positive patients in this cohort than either the first validation (37%) or training cohorts (40%), likely due to the fact that these patients tended to have larger, higher grade tumors (Table 1). The profile proved to be prognostic in both independent datasets, with a HR>10 for the first validation dataset (with the patients classified as CTC-negative having 100% 10-year DFS; Figure 1C) and a HR of 3.2 in the second validation dataset (Figure 3A). Importantly, multivariate analysis confirmed that the CTC profile was providing prognostic information independent of other clinical variables in both patient cohorts. In these patients the CTC profile in fact was a stronger predictor of outcome than measuring CTC status itself (actual CTC status HR = 3.6, *p* = 0.043 for the first independent validation cohort for which both microarray and CTC data were available), however further studies will be required to confirm this.

A study by Fan et al. [12], which used microarray expression data from the same publically-available dataset as was used in the second validation cohort in the current study, demonstrated that several prognostic profiles for breast cancer, including the Recurrence Score [6], Wound Response [5], [25], Intrinsic Subtype [7], [26], Two-Gene Ratio [27], and 70-Gene Profile (commercially available for clinical use as the MammaPrint test) [8], [9], [18], were in general highly correlated in their prognostic predictions, particularly the three most prognostic – the 70-gene, recurrence score, and wound response (*p*<0.001 by Chi-square test). Furthermore, a single model derived from all three profiles was found not to be improved compared to using any of these profiles on their own. To test whether the same was true for the CTC profile, we compared and combined it with the 70-gene profile in a second independent dataset. As previously reported [8], the 70-gene profile was highly prognostic, particularly in the first five years after diagnosis, with a 43% difference in DFS between high-risk and low-risk patients 5 years after diagnosis and a 40% difference 10 years after diagnosis (Figure 3B). Interestingly, although having less prognostic power than MammaPrint by itself, the CTC-profile was prognostic over longer survival intervals - from a 23% difference for 5-year DFS between CTC-negative and CTC-positive to 30% difference for 10-years DFS (Figure 3A). This is likely because the MammaPrint test was specifically built to identify patients at risk of early metastasis (chemotherapy sensitive), by distinguishing patients who experienced progression to metastatic disease within 5 years of diagnosis versus those who did not. Conversely the CTC profile was built under no specific follow-up time constraints. Indeed, accurately determining CTC status at diagnosis has the potential to provide prognostic information over an extended period after sampling since disseminated tumor cells have the ability to remain dormant for many years before becoming active and forming an overt growth [28].

Multivariate analyses demonstrated that the two profiles were providing prognostic information not only independent of each other, but also other common clinical variables (CTC-profile HR = 2.8 (*p*<0.05), 70-gene profile HR = 5.4 (*p*<0.0001); Table 2B): when the two profiles were combined, those patients classified as low-risk by both profiles had a 91% 10-year DFS (versus 84% for the MammaPrint and 83% for the CTC profiles alone), and those patients determined to be high-risk by both profiles had a 35% 10-year DFS (versus 44% for the MammaPrint and 53% for the CTC profiles alone). This suggests that the development of a model capturing the prognostic power of both profiles could therefore complement (particularly the specificity of) an already powerful commercially-available prognostic test for breast cancer.

When samples from this second validation cohort were divided by hormone receptor/HER2 status and into intrinsic subtypes using the gene expression data [7], those classified as CTC-positive were significantly more likely to be ER/PR/HER2-negative and therefore of the more aggressive basal intrinsic subtype. Similarly, those classified as CTC-negative were significantly more likely to be of the more indolent luminal A subtype (Pearson Chi-square analyses; data not shown). This is in contrast to the recent study of Reyal et al. [29], which found no correlation between predicted CTC status and subtype. This may be due to the small sample sized used by Reyal, et al. (with only 15 CTC-positive samples included in their study). Those patients from this cohort classified as 70-gene “high-risk” were equally likely to be either ER or PR positive or negative, and only slightly more likely to be HER2-negative. Similarly, those patients classified as both CTC-positive and 70-gene high risk (*n* = 48) were equally likely to be ER-positive or ER-negative (48% versus 52%), but significantly more likely to be HER2-negative versus HER-2 positive (63% versus 37%, p = 0.049, Pearson Chi-square), and PR-positive than PR-negative (63% versus 37%, *p*<0.031).

Functional annotation showed that, as in other studies of prognostic gene expression profiles, genes involved in cellular growth and proliferation were particularly overrepresented. In addition, gene networks associated with cellular migration and adhesion made up a large proportion of the profile (14 of 34 genes), which was expected due to the specific aim of the study. This is in contrast to our previous study [15] for which a predictive profile for tumor cell dissemination to the bone marrow was developed, in which genes associated with transport, ATP binding and regulation of transcription were overrepresented (there were in fact no genes in common between the two profiles). This may be due to the fact that dissemination to the blood versus bone marrow may to some extent represent distinct aspects of the disease, with the route of dissemination reflecting the underlying biology of the tumor. For example, we have previously shown that tumor cell dissemination to the bone marrow tends to be more clinically significant in less aggressive Luminal A type tumors, whereas dissemination to the peripheral blood tends happen more often in basal-type tumors. This would result in the genes making up the classifier being dissimilar depending on the type of dissemination that is being predicted. Further study of these genes could lead to new insights into the processes of tumor cell dissemination, which is currently imperfectly understood. None of the 6 marker genes used in the QPCR-based detection of CTCs were present in the CTC-profile, however one of the CTC-profile genes (ANKRD) was identified as a strong candidate CTC marker in a previous SAGE study undertaken by us [30]. This may also suggest that there are other novel markers for CTC detection present in the profile, which we are currently investigating. Both the growth/proliferation and migration/adhesion gene networks were important for the predictive and prognostic value of the CTC profile, and removing genes from either significantly degraded the performance of the classifier (data not shown).

In conclusion, we have developed a microarray signature that can accurately predict CTC status in breast cancer patients based on gene expression in the primary tumor, which was not only independent of other clinical variables, but also MammaPrint, a prognostic microarray test used in the clinic for breast cancer patients. In the future this may lead to a model combining both a standard prognostic microarray test and a CTC-predictive test for breast cancer and in this way not only realize a significant benefit to prognostic power, but in some circumstances also even replace blood-based prognostic CTC tests at time of diagnosis for those patients already undergoing testing by multigene assays.

## Supporting Information

Table S1 The 34 genes that make up the CTC-predictive profile, together with their cellular location, gene product type, and whether they were up- or down-regulated in tumors from CTC-positive patients. Those in **bold** were the 22 that were available to estimate the CTC profile in the first validation dataset.
(DOCX)

Table S2 Additional clinical data to accompany GEO microarray dataset GSE3985. Included is the actual CTC status based on the QPCR analysis of peripheral blood, predicted CTC status based on the microarray analysis of tumor material, relapse status (either local, loco-regional, or systemic), time to relapse (months), histological grade, HR/HER2 status (0 = negative, 1 = positive), and tumor size for the lymph node negative patients used to validate the CTC-predictive microarray signature.
(DOCX)

Table S3 Additional clinical data to accompany GEO microarray dataset GSE31364. Included is the actual CTC status based on the QPCR analysis of peripheral blood, the predicted CTC status based on the microarray analysis of tumor material, histological grade, HR status, HER2 status, and tumor size. (0 = negative, 1 = positive.)
(DOCX)

Table S4 Additional clinical data to accompany Validation Cohort 2. Included is the predicted CTC status based on the microarray analysis of tumor material, histological grade, ER/PR/HER2 status (0 = negative, 1 = positive), and tumor size for the lymph node negative patients used to validate the CTC-predictive microarray signature.
(DOCX)
