# Supplementary material for: A Prognostic Gene Expression Profile That Predicts Circulating Tumor Cell Presence in Breast Cancer Patients
Source: PLoS One. 2012 Feb 23;7(2):e32426. doi: 10.1371/journal.pone.0032426 (PMC3285692; doi:10.1371/journal.pone.0032426)
Supplement: Table S1 — The 34 genes that make up the CTC-predictive profile, together with their cellular location, gene product type, and whether they were up- or down-regulated in tumors from CTC-positive patients. Those in bold were the 22 that were available to estimate the CTC profile in the first validation dataset. (DOCX) [file pone.0032426.s001.docx]

| *Probe ID* | *Symbol* | *Entrez Gene Name* | *Cellular Location* | *Type* | *Expression in*  *CTC-positive samples* |
| --- | --- | --- | --- | --- | --- |
|  |  |  |  |  |  |
| A_32_P400570 | A1CF | APOBEC1 complementation factor | Nucleus | enzyme | Upregulated |
| **A_24_P147242** | **ABCC2** | **ATP-binding cassette, sub-family C (CFTR/MRP), member 2** | **Plasma Membrane** | **transporter** | Upregulated |
| **A_23_P66328** | **ACSM2B** | **acyl-CoA synthetase medium-chain family member 2B** | **Unknown** | **enzyme** | Upregulated |
| **A_23_P206884** | **ACSM3** | **acyl-CoA synthetase medium-chain family member 3** | **Cytoplasm** | **enzyme** | Upregulated |
| **A_23_P359043** | **AKAP2** | **A kinase (PRKA) anchor protein 2** | **Cytoplasm** | **other** | Upregulated |
| **A_24_P73577** | **ALDH1A2** | **aldehyde dehydrogenase 1 family, member A2** | **Cytoplasm** | **enzyme** | Upregulated |
| **A_23_P161218** | **ANKRD1** | **ankyrin repeat domain 1 (cardiac muscle)** | **Cytoplasm** | **transcription regulator** | Upregulated |
| A_33_P3423854 | C8B | complement component 8, beta polypeptide | Extracellular Space | other | Upregulated |
| A_24_P940656 | CDH4 | cadherin 4, type 1, R-cadherin (retinal) | Plasma Membrane | other | Upregulated |
| **A_23_P16433** | **DLL3** | **delta-like 3 (Drosophila)** | **Extracellular Space** | **other** | Upregulated |
| **A_33_P3303305** | **DSCAM** | **Down syndrome cell adhesion molecule** | **Plasma Membrane** | **other** | Upregulated |
| **A_23_P3295** | **FAM81A** | **family with sequence similarity 81, member A** | **Unknown** | **other** | Upregulated |
| **A_33_P3423969** | **GBX2** | **gastrulation brain homeobox 2** | **Nucleus** | **transcription regulator** | Upregulated |
| **A_23_P119886** | **GCKR** | **glucokinase (hexokinase 4) regulator** | **Nucleus** | **other** | Upregulated |
| **A_24_P254079** | **ICAM5** | **intercellular adhesion molecule 5, telencephalin** | **Plasma Membrane** | **other** | Upregulated |
| A_24_P404458 | ITGB1BP3 | integrin beta 1 binding protein 3 | Plasma Membrane | other | Upregulated |
| **A_23_P58419** | **KDR** | **kinase insert domain receptor (a type III receptor tyrosine kinase)** | **Plasma Membrane** | **kinase** | Downregulated |
| A_24_P649507 | MGC5566 | hypothetical protein MGC5566 | Unknown | other | Upregulated |
| A_23_P37167 | MYH6 | myosin, heavy chain 6, cardiac muscle, alpha | Cytoplasm | enzyme | Upregulated |
| A_23_P341938 | NOG | noggin | Extracellular Space | other | Upregulated |
| A_23_P168296 | NR2E1 | nuclear receptor subfamily 2, group E, member 1 | Nucleus | ligand-dependent nuclear receptor | Upregulated |
| **A_23_P82088** | **NRN1** | **neuritin 1** | **Cytoplasm** | **other** | Upregulated |
| **A_23_P502706** | **PAX3** | **paired box 3** | **Nucleus** | **transcription regulator** | Upregulated |
| **A_33_P3240018** | **PDE3B** | **phosphodiesterase 3B, cGMP-inhibited** | **Cytoplasm** | **enzyme** | Upregulated |
| A_23_P102864 | PRSS7 | protease, serine, 7 (enterokinase) | Extracellular Space | peptidase | Upregulated |
| **A_24_P235248** | **PTPRZ1** | **protein tyrosine phosphatase, receptor-type, Z polypeptide 1** | **Plasma Membrane** | **phosphatase** | Upregulated |
| **A_24_P147242** | **RBFOX1** | **RNA binding protein, fox-1 homolog (C. elegans) 1** | **Cytoplasm** | **other** | Upregulated |
| **A_32_P734012** | **RPS8** | **ribosomal protein S8** | **Cytoplasm** | **other** | Downregulated |
| **A_32_P536809** | **TEAD1** | **TEA domain family member 1 (SV40 transcriptional enhancer factor)** | **Nucleus** | **transcription regulator** | Upregulated |
| A_24_P328446 | TLX2 | T-cell leukemia homeobox 2 | Nucleus | transcription regulator | Upregulated |
| A_33_P3411632 | TMEM121 | transmembrane protein 121 | Unknown | other | Upregulated |
| A_23_P140355 | VRTN | vertebrae development homolog (pig) | Unknown | other | Upregulated |
| **A_23_P169097** | **WISP1** | **WNT1 inducible signaling pathway protein 1** | **Extracellular Space** | **other** | Downregulated |
| **A_24_P102091** | **ZDHHC21** | **zinc finger, DHHC-type containing 21** | **Plasma Membrane** | **other** | Downregulated |

***Supplmentary Table S1:***  The 34 genes that make up the CTC-predictive profile, together with their cellular location, gene product type, and whether they were up- or down-regulated in tumors from CTC-positive patients. Those in **bold** were the 22 that were available to estimate the CTC profile in the first validation dataset.
