# Supplementary material for: A Prognostic Gene Expression Profile That Predicts Circulating Tumor Cell Presence in Breast Cancer Patients
Source: PLoS One. 2012 Feb 23;7(2):e32426. doi: 10.1371/journal.pone.0032426 (PMC3285692; doi:10.1371/journal.pone.0032426)
Supplement: Table S2 — Additional clinical data to accompany GEO microarray dataset GSE3985. Included is the actual CTC status based on the QPCR analysis of peripheral blood, predicted CTC status based on the microarray analysis of tumor material, relapse status (either local, loco-regional, or systemic), time to relapse (months), histological grade, HR/HER2 status (0 = negative, 1 = positive), and tumor size for the lymph node negative patients used to validate the CTC-predictive microarray signature. (DOCX) [file pone.0032426.s002.docx]

| Sample ID | CTC Status (QPCR) | CTC Status (Microarray) | Relapse | Time to Relapse | Histological Grade | HR Status | HER2 Status | Tumor Size |
| --- | --- | --- | --- | --- | --- | --- | --- | --- |
| 14 | 1 | 1 | 0 | 104.41 | 2 | 1 | 2 | T0 |
| 20 | 1 | 1 | 0 | 81.68 | 2 | 2 | 1 | T0 |
| 24 | 1 | 1 | 1 | 54.08 | 2 | 1 | 1 | T1 |
| 31 | 1 | 0 | 1 | 10.03 | 3 | 2 |  | T0 |
| 34 | 1 | 1 | 1 | 27.37 | 2 | 2 | 1 | T0 |
| 53 | 0 | 1 | 0 | 112.4 | 2 | 2 | 2 | T1 |
| 91 | 0 | 0 | 0 | 107.73 | 3 | 1 | 2 | T1 |
| 101 | 0 | 1 | 0 | 99.08 | 2 | 1 | 1 | T1 |
| 106 | 0 | 1 | 0 | 108.82 | 2 | 1 | 1 | T1 |
| 122 | 0 | 1 | 1 | 58.75 | 2 | 1 | 1 | T0 |
| 130 | 0 | 0 | 0 | 93.75 | 2 | 1 |  | T0 |
| 148 | 0 | 1 | 0 | 106.91 | 3 | 1 | 1 | T1 |
| 150 | 0 | 0 | 0 | 110.79 | 2 | 1 | 1 | T0 |
| 157 | 0 | 0 | 0 | 97.07 | 2 | 1 | 2 | T0 |
| 165 | 0 | 0 | 0 | 101.84 | 2 | 2 | 2 | T0 |
| 171 | 0 | 0 | 0 | 108.49 | 1 | 1 | 2 | T0 |
| 197 | 0 | 0 | 0 | 90.66 | 3 | 2 | 1 | T0 |
| 210 | 0 | 0 | 0 | 106.61 | 2 | 1 | 1 | T0 |
| 212 | 0 | 0 | 0 | 108.88 | 1 | 1 | 1 | T0 |
| 223 | 0 | 0 | 0 | 43.19 | 2 | 1 | 1 | T1 |
| 232 | 0 | 0 | 0 | 105.33 | 3 | 1 | 1 | T0 |
| 234 | 0 | 1 | 0 | 104.97 | 2 | 1 | 1 | T1 |
| 240 | 0 | 0 | 0 | 102.5 | 1 |  |  | T0 |
| 246 | 0 | 0 | 0 | 98.98 | 3 | 2 | 1 | T1 |
| 247 | 0 | 0 | 0 | 88.82 | 3 | 2 | 1 | T0 |
| 264 | 0 | 0 | 0 | 55.43 | 1 | 1 | 1 | T0 |
| 275 | 0 | 1 | 0 | 103.45 | 2 | 1 | 1 | T1 |
| 277 | 0 | 0 | 0 | 94.84 | 2 | 1 | 1 | T0 |
| 293 | 1 | 0 | 1 | 46.91 | 3 | 2 | 2 | T0 |
| 300 | 0 | 0 | 0 | 101.02 | 2 | 1 | 1 | T1 |
| 314 | 0 | 0 | 0 | 86.58 | 2 | 1 | 1 | T0 |
| 318 | 0 | 0 | 0 | 92.73 | 3 | 2 | 2 | T0 |
| 321 | 0 | 0 | 0 | 99.47 | 3 | 2 | 1 | T0 |
| 338 | 0 | 1 | 0 | 107.17 | 2 | 2 | 1 | T0 |
| 341 | 0 | 0 | 0 | 84.93 | 1 | 1 | 1 | T0 |
| 355 | 0 | 0 |  |  | 2 | 1 | 1 | T0 |
| 357 | 0 | 1 | 0 | 102.2 | 2 | 1 | 1 | T1 |
| 363 | 0 | 0 | 0 | 89.77 | 2 | 1 | 1 | T0 |
| 371 | 0 | 0 | 1 | 15.56 | 3 | 2 | 1 | T0 |
| 373 | 0 | 1 | 0 | 101.25 | 2 | 2 | 1 | T0 |
| 381 | 0 | 1 | 0 | 95.39 | 1 | 1 | 1 | T0 |
| 388 | 0 | 1 | 0 | 85.79 | 1 | 1 | 1 | T0 |
| 406 | 0 | 1 | 1 | 62.66 | 2 | 1 | 1 | T1 |
| 430 | 0 | 1 | 0 | 94.14 | 2 | 1 | 1 | T1 |
| 470 | 0 | 0 | 0 | 85.39 | 2 | 1 | 1 | T0 |
| 539 | 0 | 0 | 0 | 90.07 | 1 | 2 | 1 | T1 |
| 551 | 0 | 0 | 0 | 27.83 | 3 | 2 | 1 | T1 |
| 709 | 0 | 0 | 1 | 8.45 | 3 | 2 | 1 | T1 |
| 722 | 0 | 0 | 0 | 82.37 | 2 | 1 | 1 | T1 |

***Supplementary Table S2:*** Additional clinical data to accompany GEO microarray dataset GSE3985. Included is the actual CTC status based on the QPCR analysis of peripheral blood, predicted CTC status based on the microarray analysis of tumor material, relapse status (either local, loco-regional, or systemic), time to relapse (months), histological grade, HR/HER2 status (0 = negative, 1 = positive), and tumor size for the lymph node negative patients used to validate the CTC-predictive microarray signature.
