# Supplementary material for: A Prognostic Gene Expression Profile That Predicts Circulating Tumor Cell Presence in Breast Cancer Patients
Source: PLoS One. 2012 Feb 23;7(2):e32426. doi: 10.1371/journal.pone.0032426 (PMC3285692; doi:10.1371/journal.pone.0032426)
Supplement: Table S3 — Additional clinical data to accompany GEO microarray dataset GSE31364. Included is the actual CTC status based on the QPCR analysis of peripheral blood, the predicted CTC status based on the microarray analysis of tumor material, histological grade, HR status, HER2 status, and tumor size. (0 = negative, 1 = positive.) (DOCX) [file pone.0032426.s003.docx]

| Sample ID | CTC Status (QPCR) | CTC Status (Microarray) | Histological Grade | HR Status | HER2 Status | Tumor Size |
| --- | --- | --- | --- | --- | --- | --- |
| 6001030 | 1 | 1 | 2 | 1 | 0 | T3 |
| 6001035 | 1 | 1 | 3 | 1 | 0 | T1 |
| 6001043 | 1 | 1 | 1 | 0 | 1 | T3 |
| 6001044 | 1 | 1 | 3 | 1 | 0 | T1 |
| 6001760 | 1 | 1 | 2 | 1 | 1 | T3 |
| 6001761 | 0 | 0 | 3 | 0 | 1 | T2 |
| 6001762 | 1 | 0 | 2 | 1 | 0 | T1 |
| 6001763 | 1 | 0 | 2 | 1 | 0 | T1 |
| 6001764 | 0 | 0 | 2 | 1 | 1 | T1 |
| 6001765 | 0 | 0 | 2 | 1 | 0 | T2 |
| 6001766 | 1 | 1 | 3 | 1 | 0 | T3 |
| 6001767 | 1 | 1 | 1 | 0 | 0 | T1 |
| 6001768 | 1 | 1 | 2 | 1 | 0 | T1 |
| 6001769 | 1 | 1 | 3 | 1 | 1 | T3 |
| 6001770 | 1 | 1 | 3 | 1 | 0 | T2 |
| 6001842 | 1 | 1 | 3 | 0 | 0 | T1 |
| 6001843 | 1 | 1 | 3 | 0 | 0 | T1 |
| 6001844 | 0 | 1 | 2 | 1 | 0 | T1 |
| 6001845 | 0 | 1 | 3 | 0 | 0 | T2 |
| 6001846 | 0 | 0 | 2 | 1 | 0 | T1 |
| 6001847 | 0 | 1 | 3 | 0 | 1 | T2 |
| 6001848 | 0 | 1 | 1 | 1 | 0 | T1 |
| 6001849 | 1 | 0 | 1 | 1 | 0 | T1 |
| 6001850 | 0 | 0 | 2 | 1 | 0 | T1 |
| 6001851 | 0 | 0 | 2 | 1 | 1 | T1 |
| 6001852 | 0 | 0 | 2 | 1 | 0 | T1 |
| 7000427 | 1 | 0 | 2 | 1 | 0 | T2 |
| 7000428 | 0 | 0 | 2 | 1 | 0 | T1 |
| 7000429 | 0 | 0 | 2 | 0 | 0 | T1 |
| 7000431 | 0 | 0 | 2 | 1 | 1 | T2 |
| 7000432 | 0 | 0 | 1 | 1 | 0 | T1 |
| 7000433 | 0 | 0 | 1 | 1 | 0 | T1 |
| 7000434 | 0 | 0 | 1 | 1 | 0 | T1 |
| 7000435 | 1 | 0 | 3 | 0 | 1 | T2 |
| 7000436 | 0 | 0 | 2 | 1 | 0 | T1 |
| 7000438 | 0 | 0 | 2 | 1 | 0 | T1 |
| 7000439 | 0 | 0 | 2 | 1 | 0 | T2 |
| 7000440 | 0 | 0 | 2 | 1 | 0 | T1 |
| 7000441 | 0 | 1 | 3 | 1 | 0 | T1 |
| 7000442 | 0 | 0 | 2 | 1 | 0 | T1 |
| 7000443 | 0 | 0 | 1 | 1 | 0 | T1 |
| 7000444 | 0 | 0 | 1 | 1 | 0 | T1 |
| 7000445 | 0 | 0 | 3 | 1 | 1 | T1 |
| 7000446 | 0 | 0 | 2 | 1 | 0 | T1 |
| 7000447 | 0 | 0 | 2 | 1 | 0 | T2 |
| 7000449 | 0 | 0 | 2 | 1 | 0 | T1 |
| 7000450 | 1 | 0 | 3 | 1 | 0 | T2 |
| 7000451 | 0 | 0 | 1 | 1 | 1 | T1 |
| 7000452 | 0 | 0 | 2 | 1 | 0 | T1 |
| 7000454 | 0 | 0 | 2 | 1 | 0 | T1 |
| 7000455 | 0 | 0 | 3 | 0 | 0 | T2 |
| 7000456 | 0 | 0 | 2 | 1 | 1 | T2 |
| 7000457 | 0 | 0 | 2 | 1 | 0 | T1 |
| 7000458 | 0 | 0 | 1 | 1 | 0 | T1 |
| 7000459 | 0 | 0 | 3 | 1 | 1 | T1 |
| 7000460 | 0 | 0 | 1 | 0 | 0 | T1 |
| 7000461 | 0 | 0 | 2 | 1 | 0 | T1 |
| 7000463 | 1 | 0 | 3 | 1 | 1 | T1 |
| 7000465 | 0 | 0 | 2 | 1 | 0 | T3 |
| 7000467 | 0 | 0 | 3 | 1 | 0 | T1 |
| 7000480 | 1 | 1 | 2 | 0 | 0 | T1 |
| 7000481 | 1 | 1 |  |  |  | T1 |
| 7000482 | 1 | 0 | 2 | 1 | 0 | T2 |
| 7001514 | 1 | 1 | 2 | 1 | 0 | T1 |
| 7001515 | 1 | 1 | 1 | 1 | 0 | T3 |
| 7001516 | 1 | 1 | 1 | 1 | 0 | T2 |
| 7001519 | 1 | 1 | 2 | 1 | 1 | T1 |
| 7001520 | 1 | 1 |  |  |  | T3 |
| 7001521 | 1 | 1 | 2 | 1 | 0 | T1 |
| 7001523 | 1 | 1 | 1 | 1 | 0 | T2 |
| 7001524 | 1 | 1 | 2 | 1 | 1 | T1 |
| 7001525 | 1 | 1 | 2 | 1 | 0 | T2 |

***Supplementary Table S3:*** Additional clinical data to accompany GEO microarray dataset GSE31364. Included is the actual CTC status based on the QPCR analysis of peripheral blood, the predicted CTC status based on the microarray analysis of tumor material, histological grade, HR status, HER2 status, and tumor size. (0 = negative, 1 = positive.)
