# Supplementary material for: A Prognostic Gene Expression Profile That Predicts Circulating Tumor Cell Presence in Breast Cancer Patients
Source: PLoS One. 2012 Feb 23;7(2):e32426. doi: 10.1371/journal.pone.0032426 (PMC3285692; doi:10.1371/journal.pone.0032426)
Supplement: Table S4 — Additional clinical data to accompany Validation Cohort 2. Included is the predicted CTC status based on the microarray analysis of tumor material, histological grade, ER/PR/HER2 status (0 = negative, 1 = positive), and tumor size for the lymph node negative patients used to validate the CTC-predictive microarray signature. (DOCX) [file pone.0032426.s004.docx]

| Sample ID | CTC Status (Microarray) | 70-Gene Profile | Histological Grade | ER Status | PR Status | HER2 Status | Tumor Size |
| --- | --- | --- | --- | --- | --- | --- | --- |
| 4 | 1 | 1 | 3 | 0 | 0 |  | T1 |
| 6 | 0 | 0 | 2 | 0 | 0 | 0 | T1 |
| 7 | 0 | 0 | 1 | 0 | 0 | 0 | T1 |
| 8 | 1 | 1 | 3 | 1 | 1 | 0 | T2 |
| 9 | 1 | 0 | 3 | 0 | 0 | 0 | T1 |
| 11 | 0 | 0 | 3 | 0 | 0 | 1 | T2 |
| 13 | 0 | 1 | 3 | 0 | 0 | 1 | T2 |
| 14 | 0 | 0 | 1 | 0 | 0 | 0 | T1 |
| 17 | 0 | 0 | 2 | 0 | 0 | 0 | T1 |
| 26 | 0 | 0 | 3 | 0 | 0 |  | T1 |
| 27 | 1 | 0 | 1 | 0 | 0 | 0 | T1 |
| 29 | 1 | 0 | 2 | 0 | 0 | 0 | T1 |
| 38 | 0 | 1 | 2 | 0 | 0 | 0 | T1 |
| 39 | 1 | 0 | 3 | 0 | 0 | 0 | T2 |
| 45 | 1 | 0 | 3 | 0 | 0 | 0 | T2 |
| 48 | 1 | 1 | 3 | 1 | 1 | 0 | T2 |
| 57 | 1 | 1 | 3 | 1 | 1 | 0 | T2 |
| 58 | 1 | 1 | 3 | 0 | 0 | 0 | T2 |
| 59 | 1 | 1 | 3 | 0 | 1 | 0 | T2 |
| 61 | 0 | 1 | 3 | 0 | 0 | 0 | T1 |
| 62 | 1 | 1 | 3 | 0 | 1 | 1 | T2 |
| 72 | 0 | 1 | 2 | 0 | 0 | 0 | T2 |
| 73 | 1 | 1 | 3 | 0 | 1 | 0 | T2 |
| 75 | 1 | 1 | 3 | 1 | 1 | 0 | T2 |
| 76 | 0 | 1 | 2 | 1 | 1 | 1 | T2 |
| 103 | 1 | 1 | 3 | 1 | 1 |  | T2 |
| 107 | 1 | 1 | 3 | 0 | 0 | 0 | T1 |
| 111 | 1 | 1 | 3 | 0 | 0 |  | T2 |
| 117 | 1 | 0 | 1 | 0 | 0 | 0 | T1 |
| 118 | 0 | 0 | 2 | 0 | 0 | 0 | T1 |
| 122 | 1 | 0 | 2 | 0 | 0 | 0 | T2 |
| 123 | 0 | 1 | 3 | 0 | 0 | 0 | T1 |
| 124 | 0 | 0 | 2 | 0 | 0 |  | T1 |
| 126 | 1 | 0 | 3 | 0 | 0 | 0 | T1 |
| 131 | 1 | 1 | 3 | 1 | 1 | 0 | T1 |
| 133 | 0 | 0 | 1 | 0 | 0 | 0 | T1 |
| 139 | 1 | 1 | 3 | 0 | 0 | 0 | T2 |
| 140 | 1 | 0 | 1 | 0 | 0 |  | T1 |
| 142 | 0 | 0 | 3 | 0 | 0 | 1 | T1 |
| 144 | 1 | 1 | 3 | 1 | 1 | 0 | T2 |
| 148 | 1 | 1 | 3 | 1 | 1 | 1 | T2 |
| 153 | 1 | 1 | 3 | 1 | 0 | 1 | T2 |
| 154 | 1 | 0 | 1 | 0 | 0 | 0 | T1 |
| 155 | 1 | 0 | 3 | 0 | 0 | 0 | T2 |
| 165 | 1 | 1 | 3 | 0 | 0 | 0 | T1 |
| 167 | 1 | 0 | 1 | 0 | 0 | 1 | T1 |
| 175 | 1 | 1 | 2 | 0 | 0 |  | T1 |
| 179 | 0 | 1 | 3 | 0 | 1 | 0 | T2 |
| 181 | 0 | 1 | 3 | 1 | 1 | 1 | T1 |
| 183 | 1 | 0 | 1 | 0 | 0 | 0 | T2 |
| 185 | 0 | 1 | 2 | 0 | 0 | 0 | T1 |
| 189 | 1 | 1 | 2 | 1 | 1 | 1 | T2 |
| 191 | 0 | 0 | 3 | 1 | 1 | 0 | T2 |
| 193 | 0 | 0 | 1 | 0 | 0 | 0 | T1 |
| 199 | 0 | 0 | 3 | 0 | 0 | 1 | T1 |
| 201 | 0 | 0 | 3 | 0 | 0 | 0 | T2 |
| 202 | 1 | 1 | 3 | 1 | 1 | 1 | T2 |
| 205 | 0 | 1 | 3 | 0 | 0 | 0 | T1 |
| 207 | 1 | 0 | 1 | 0 | 0 | 0 | T2 |
| 212 | 1 | 1 | 2 | 1 | 1 | 0 | T1 |
| 215 | 1 | 1 | 3 | 1 | 1 | 0 | T2 |
| 219 | 0 | 0 | 1 | 0 | 0 | 0 | T1 |
| 222 | 1 | 1 | 3 | 1 | 1 | 0 | T1 |
| 224 | 0 | 1 | 1 | 0 | 0 | 0 | T1 |
| 227 | 1 | 1 | 3 | 0 | 0 | 0 | T1 |
| 229 | 1 | 1 | 3 | 0 | 0 | 1 | T2 |
| 230 | 1 | 1 | 3 | 1 | 1 | 1 | T1 |
| 233 | 0 | 0 | 1 | 0 | 0 | 0 | T2 |
| 235 | 1 | 0 | 1 | 0 | 0 | 0 | T1 |
| 239 | 1 | 0 | 1 | 0 | 0 | 0 | T1 |
| 240 | 1 | 1 | 3 | 0 | 0 | 0 | T2 |
| 241 | 1 | 1 | 3 | 1 | 1 | 0 | T2 |
| 246 | 0 | 0 | 3 | 1 | 1 |  | T1 |
| 251 | 0 | 1 | 3 | 0 | 0 | 1 | T1 |
| 254 | 0 | 1 | 3 | 0 | 0 | 0 | T2 |
| 259 | 1 | 0 | 1 | 0 | 0 | 0 | T1 |
| 266 | 0 | 0 | 1 | 0 | 0 | 1 | T1 |
| 268 | 1 | 1 | 3 | 1 | 1 | 0 | T2 |
| 270 | 1 | 1 | 3 | 1 | 1 | 0 | T2 |
| 271 | 0 | 0 | 1 | 0 | 0 | 0 | T1 |
| 274 | 0 | 1 | 1 | 0 | 1 | 0 | T1 |
| 278 | 1 | 0 | 2 | 0 | 0 | 0 | T2 |
| 285 | 1 | 0 | 2 | 0 | 0 | 0 | T2 |
| 286 | 1 | 1 | 2 | 0 | 1 | 1 | T1 |
| 295 | 1 | 0 | 1 | 0 | 0 | 0 | T2 |
| 296 | 1 | 1 | 3 | 0 | 0 | 1 | T2 |
| 302 | 1 | 1 | 3 | 0 | 0 | 0 | T2 |
| 304 | 0 | 0 | 1 | 0 | 0 | 0 | T1 |
| 305 | 1 | 0 | 1 | 0 | 0 | 0 | T2 |
| 306 | 1 | 0 | 2 | 0 | 0 | 0 | T1 |
| 312 | 1 | 0 | 2 | 0 | 0 | 0 | T2 |
| 313 | 0 | 0 | 2 | 0 | 0 | 0 | T1 |
| 317 | 1 | 1 | 2 | 0 | 1 | 0 | T1 |
| 319 | 1 | 1 | 3 | 1 | 0 | 1 | T2 |
| 323 | 1 | 0 | 1 | 0 | 0 | 0 | T2 |
| 326 | 1 | 1 | 3 | 1 | 1 | 0 | T2 |
| 329 | 1 | 1 | 1 | 0 | 0 | 0 | T2 |
| 331 | 1 | 1 | 1 | 0 | 0 | 0 | T1 |
| 333 | 1 | 1 | 3 | 1 | 1 | 1 | T2 |
| 336 | 0 | 0 | 1 | 0 | 0 | 0 | T1 |
| 338 | 1 | 1 | 3 | 1 | 1 | 0 | T1 |
| 339 | 1 | 0 | 2 | 0 | 0 | 0 | T1 |
| 342 | 0 | 0 | 2 | 0 | 0 | 0 | T1 |
| 344 | 1 | 0 | 1 | 0 | 0 | 0 | T1 |
| 348 | 1 | 0 | 2 | 0 | 0 | 0 | T1 |
| 349 | 1 | 0 | 2 | 0 | 0 | 0 | T2 |
| 352 | 1 | 0 | 2 | 0 | 0 | 0 | T2 |
| 354 | 0 | 0 | 3 | 0 | 0 | 0 | T2 |
| 356 | 0 | 0 | 1 | 0 | 0 | 0 | T1 |
| 365 | 0 | 0 | 2 | 0 | 0 | 0 | T1 |
| 366 | 0 | 1 | 2 | 0 | 1 | 0 | T1 |
| 367 | 1 | 1 | 2 | 0 | 0 | 1 | T2 |
| 368 | 1 | 0 | 2 | 0 | 0 | 0 | T2 |
| 369 | 1 | 1 | 2 | 1 | 1 | 1 | T1 |
| 371 | 1 | 1 | 2 | 0 | 1 | 0 | T1 |
| 379 | 1 | 1 | 1 | 0 | 1 | 0 | T2 |
| 380 | 1 | 0 | 2 | 0 | 0 | 0 | T1 |
| 388 | 1 | 0 | 2 | 0 | 0 | 0 | T1 |
| 391 | 1 | 0 | 2 | 0 | 1 | 0 | T2 |
| 394 | 1 | 0 | 2 | 0 | 0 | 0 | T1 |
| 397 | 0 | 1 | 2 | 0 | 0 | 0 | T2 |
| 398 | 1 | 1 | 3 | 1 | 1 | 0 | T2 |

***Supplementary Table S4:*** Additional clinical data to accompany Validation Cohort 2. Included is the predicted CTC status based on the microarray analysis of tumor material, histological grade, ER/PR/HER2 status (0 = negative, 1 = positive), and tumor size for the lymph node negative patients used to validate the CTC-predictive microarray signature.
